# Fish under pressure: Examining behavioural responses of Iberian barbel under simulated hydropeaking with instream structures

**DOI:** 10.1371/journal.pone.0211115

**Published:** 2019-01-23

**Authors:** M. J. Costa, J. F. Fuentes-Pérez, I. Boavida, J. A. Tuhtan, A. N. Pinheiro

**Affiliations:** 1 Civil Engineering Research and Innovation for Sustainability, Instituto Superior Técnico, Universidade de Lisboa, Lisboa, Portugal; 2 Centro de Estudos Florestais, Instituto Superior de Agronomia, Universidade de Lisboa, Lisboa, Portugal; 3 Centre for Biorobotics, Tallinn University of Technology, Tallinn, Estonia; University of Windsor, CANADA

## Abstract

Hydropeaking is the rapid change in the water flow downstream of a hydropower plant, driven by changes in daily electricity demand. These fluctuations may produce negative effects in freshwater fish. To minimize these impacts, previous studies have proposed habitat enhancement structures as potential mitigation measures for salmonids. However, the recommendation of these mitigation measures for cyprinids remains scarce and their effects unknown. In this study, the effects of potential habitat mitigation structures under simulated hydropeaking and base-flow conditions are examined for Iberian barbel (*Luciobarbus bocagei*) in an indoor flume. Solid triangular pyramids and v-shaped structures were evaluated as potential flow-refuging areas and compared with a configuration without structures. A novel, interdisciplinary approach is applied to investigate individual and group responses to rapidly changing flows, by assessing physiological (glucose and lactate), movement behaviour (structure use, sprints and drifts) and the pressure distribution using a fish-inspired artificial lateral line flow sensor. The major findings of this study are four-fold: 1) Under hydropeaking conditions, the v-shaped structures triggered a lactate response and stimulated individual structure use, whereas solid structures did not elicit physiological adjustments and favoured individual and group structure use. Overall, both solid structures and their absence stimulated sprints and drifts. 2) The hydrodynamic conditions created in hydropeaking did not always reflect increased physiological responses or swimming activity. 3) Each event-structure combination resulted in unique hydrodynamic conditions which were reflected in the different fish responses. 4) The most relevant flow variable measured was the pressure asymmetry, which is caused by the vortex size and shedding frequency of the structures. Considering the non-uniform nature of hydropeaking events, and the observation that the fish responded differently to specific flow event-structure combinations, a diverse set of instream structures should be considered for habitat-based hydropeaking mitigation measures for Iberian barbel.

## 1. Introduction

Hydropower provides an immediate and renewable source of electricity capable of responding to rapid daily fluctuations in electricity network demand. Accordingly, the construction of hydropower plants has continued worldwide [1,2]. Due to the high fluctuations in daily electricity demand, the controlled discharge of water through the turbines creates hydropeaking, manifested as rapid flow fluctuations in the receiving water [3]. The rapid flow fluctuations result from the distinct phases of hydropeaking: base-flow discharge (no electricity production), increasing discharge or up-ramping (rapid increase in electricity production), continuous high peak discharge (peak energy demand), and decreasing discharge or down-ramping (shutdown of the turbines) [4]. The range of flow alterations depends highly on the operation scheme of the hydropower plant. In regulated rivers having hydropower plants with inter-annual storage capacity, the peak-flows can exceed the base-flow by a factor of eight [5]. The effects of these changes can be particularly noticeable in Iberian rivers affected by Mediterranean climate, where in summer there is low water availability in comparison with winter, and the environmental flows cannot exceed those of the natural flow regime [6]. For example, during summer periods the flow ratio can be eightfold, the daily peak frequency twofold, and peak duration one to two hours [7]. These fluctuations affect the ecological integrity of river ecosystems [8,9] by changing the downstream morphological and hydrological processes [10–15]. Specifically addressing freshwater fish, those changes will affect diel activities (e.g. predator avoidance, foraging or finding refuge) and life-cycle events (e.g. reproductive migratory cues, survival or growth). These activities are intrinsically associated with movement behaviour shifts which differ from those occurring within natural flow conditions (for example, in magnitude and distance covered). Thus, fish are forced to adapt, and the energetic resources necessary to complete those events may be reduced [16]. Due to their high mobility in the river system, fish reflect the longitudinal gradient of the river continuum. Thus, the biological impacts of hydropeaking have been mainly studied considering fish as the indicator organism [10]. However, extreme macroinvertebrate drifts [17] and physiological and physical constraints in riparian plants [10] have been documented.

Fish responses range from sub-organismal (e.g. neuroendocrine or metabolic adjustments) [16,18,19], to changes in key-life events [20–23]. Sub-organismal responses follow a neuroendocrine pathway, initiated by the stimulation of the hypothalamic-pituitary-interrenal axis, which aim to restore the homeostatic state [24]. Negative effects occur when the organism is no longer capable of maintaining or recovering to the homeostatic state, with repercussions on key life-cycle events, such as reproduction, growth, and survival [24,25]. To consider the broad range of biological responses, several experimental approaches have been adopted to study the effects of hydropeaking. Artificial channel [16,26–28] and *in situ* river experiments [18,19,29,30] have been conducted to investigate hydropeaking flow pulses and study the resulting fish behaviour. However, the direction and strength of specific flow-behaviour causal pathways may be identified by constraining external environmental factors under laboratory settings. Both in artificial channels and in natural conditions, transient cortisol elevations [16,19], low lactate levels [18], and insignificant glucose changes [16,18] have been reported. In artificial channels, the cortisol changes were attributed to the induced flow fluctuations [16] whereas in natural conditions they were attributed to routine physiological processes, rather than to hydropeaking [19]. In most cases, it has been difficult to establish a causal relation between flow variability and potential stress responses. Reported explanations for this difficulty include the time from stimulus perception to sample collection, the effects of other environmental and biological factors, and the experimental conditions (laboratory *vs*. field experiments) [31]. These studies suggest that hydropeaking can trigger elevations in physiological responses. However, the direction and range of responses is difficult to determine. Nonetheless, it is expected that the physiological responses under hydropeaking conditions will be higher than those in base-flow conditions.

Changes in locomotor activity are detectable as immediate responses to rapid flow fluctuations. In rivers affected by hydropeaking, these changes can be manifested as local [18,30,32–34] or large-scale spatial scale-movements [20,35–38]. However, no changes in the movement behaviour [39], as well as changes which were difficult to interpret [40] have also been reported. The diversity of movement behaviour responses has been largely attributed to physical and biological factors that were not possible to control, such as the presence of velocity refuges, and inter and intra-individual variability [18,30]. In addition to these, other physical (e.g. water quality, substrate, sediment dynamics, and hyporheic flow) and biological factors (e.g. species interactions) also affect fish movement behaviour and are difficult to reproduce in experimental flumes [41]. It is also important to note that laboratory studies cannot reproduce the full range of physical and biological factors driving fish behaviour as they exist in a river system. Nevertheless, indoor experiments have demonstrated the ability to study small-scale behavioural changes because of the possibility to control external confounding factors [42]. For example, simulated single peak events, consisting of an up-ramping stage followed by a continuous high peak, caused movement behaviour changes ranging from downstream displacement [28] to significant use of refuges [43]. This was in contrast to experiments with up- and down-ramping events, which did not find significant movement behaviour alterations [44,45]. Under controlled conditions it is possible to visualize the behavioural diversity found in nature, however it is challenging to find ecological consequences for such behaviours [46].

Only a handful of studies have investigated habitat enhancement structures based on fish behavioural changes to simulated rapidly changing flows. Lateral refuges were effectively used by Iberian barbels for flow-refuging in simulated hydropeaking conditions [27]. T-shaped structures [47] and lateral refuges [43] were suggested as potential flow-refuging areas for brown trout subjected to simulated hydropeaking. Although [48] demonstrated that brown trout used the lateral refuges for flow-refuging, [47] reported high site fidelity of juveniles of this species, rather than using the refuge areas to compensate for the fluctuating flows. Similar behaviour was also described by [49] for Atlantic salmon parr subjected to increased discharges in experimental flumes. The authors suggested that the short duration of the simulated flow events and the low habitat heterogeneity offered by artificial flumes effectively reduced the possibility of salmonid species to exhibit their full potential of movement behaviour patterns [47,49]. Previous studies on the effects of hydropeaking are focused mainly on salmonid species, whereas behavioural changes of cyprinids have been scarcely studied.

A major challenge of all studies addressing rapid flow fluctuations and their impact on fish behaviour is the difficulty to attribute a behavioural response to a specific flow fluctuation. This mechanistic link is also difficult to find because the current methods to measure the flow field are based on point values or spatial distributions of flow variables which do not take into account the inherent fluid-body interaction between a fish and the surrounding flow field. Fish experience the surrounding flow via their octavolateralis afferent system, consisting of the lateral line and inner ear sense organs [50]. The lateral line consists of a linear array of mechanoreceptors located along the body, allowing fish to sense the spatial gradients of the flow field [51]. For the majority of freshwater fish species, sensory cues correspond to near-body fluctuations ranging from 1–150 Hz [52,53]. This range covers natural hydrodynamic variability and enables fish to perceive the different spatiotemporal scales of flow, its alterations, and its interaction to the hydrodynamic environment. In this work, we attempt to characterize the hydrodynamic conditions resulting from a continuous peak flow and the presence of structures, by using the principles of the mechanosensory system of fish, through an artificial lateral line probe (LLP). To date, the assessment of fish behaviour according to the changes in the hydraulic environment has relied solely on conventional point flow measurements [54–56]. Such measurements only consider a single point in space, and rely on the velocity and corresponding turbulence metrics measured using an acoustic Doppler velocimeter or propeller. Point-based metrics neglect the fluid-body interactions which fish actually use to sense the flow, and the frequency range of velocity measurements most commonly spans 1–50 Hz, which at best covers the lower third of the sensory range of biological lateral lines [57]. Artificial lateral lines probes (LLPs) have emerged as a potential solution to fill this measurement gap. These devices consist of streamlined bodies with electromechanical sensor arrays. In this work, we investigate the potential utility of pressure sensor based LLPs. The LLP measures the rapid flow changes (up to 200 Hz) around its body using six differential pressure sensors. The motivation of using a LLP is that it more closely mimics the spatial and temporal sensing capacity of fish in contrast with low-frequency, point measurement devices [58,59]. In this study, the flow metrics derived from the LLP were based on previous work comparing the sensor data to fish behaviour in a vertical slot fishway, where the most significant variables were the mean pressure, the mean front fluctuations, and the mean pressure asymmetry [60].

The aim of this study is to assess the physiological and behavioural responses of Iberian barbel (*Luciobarbus bocagei* Steindachner, 1864) (hereafter *L*. *bocagei*) associated with simulated hydropeaking in the presence or absence of instream structures in an indoor flume. Three hypotheses were investigated: 1) Rapid flow changes trigger physiological responses in *L*. *bocagei*. 2) Movement behaviour is affected by, and differs between simulated rapid flow changes and constant flow events with instream structures. 3) Critical thresholds of local hydrodynamic variables exist for *L*. *bocagei*, and are associated with distinct behavioural responses.

## 2. Methods

### 2.1 Ethics statement

All procedures involving animal manipulation, from capture in their natural environment to holding in the laboratory, were carried out in strict accordance with European standards [46] and Portuguese protocols [47] that assure animal welfare. Fish capture, handling, transportation and holding permits were issued by the Institute for Conservation of Nature and Forests (ICNF) (permit numbers 290/2016/CAPT and 291/2016/CAPT). These permits also authorized the Laboratory of Hydraulics and Environment to hold *L*. *bocagei* in captivity for no longer than 10 days in accordance with the methodology described for this study. The experiments were conducted according to strict recommendations for the ‘‘protection of animal use for experimental and scientific work” (n° 5, article n° 31, Decree-Law 113/2013, 7th of August, transposing the European Directive n.° 2010/63/UE). In accordance with this Decree-Law, specific permits were issued by the nominated competent authority, the Direção-Geral de Alimentação e Veterinária (DGAV) (point a), article n° 3), which authorized IB and MJC to perform animal manipulation and procedures that involved animal health and welfare, and the conceptualization of projects that involved animal manipulation (n° 2, article n° 31). After revising the above-mentioned documents, the Ethics Committee of Instituto Superior Técnico (EC-IST) has given approval to carry out the experiments in the Laboratory of Hydraulics (Ref. n°6/2018 (CE-IST)). Thus, all the necessary procedures to complete this study were authorized and performed with minimum handling stress. No fish were sacrificed for the purpose of this study.

### 2.2 Fish sampling and handling

The Iberian barbel (*Luciobarbus bocagei* Steindachner, 1864) was selected to study the physiological and behavioural responses to simulated hydropeaking. Little is known about the physiological responses to flow variability by *L*. *bocagei*. However, recent studies have reported increased in glucose levels when *L*. *bocagei* was subjected to simulated hydropeaking [27,61], as well as differences in lactate levels associated with successful fishway passage between seasons [62]. This endemic cyprinid of the Iberian Peninsula is widely distributed in the river basins of northern and central Portugal [63] (IUCN status: least concern). *L*. *bocagei* is a bottom-oriented, potamodromous cyprinid [64,65], feeding continuously during the day on benthic invertebrates and plant material, and adapting its diet according to the available food resources [66,67]. Fingerlings and juveniles are predominantly rheophilic [68,69], as are the spawning adults, which prefer fast-moving currents during upstream migrations [70]. Outside the reproductive season, the adults of *L*. *bocagei* tend to be limnophilic. In this study, young adults of *L*. *bocagei* were used because they are adapted to fast-moving flow conditions Their habitat preferences change with ontogeny; juveniles prefer sand-gravel substrates and shallower areas, whereas adults prefer deeper areas with available refuges [68].

Fish were obtained at the Sorraia River (39.011376 ° N, -8.357126 ° W), a tributary of the Tagus River (central Portugal). The selected site is not affected by hydropeaking, which makes it a suitable source of unconditioned fish to study the impacts of hydropeaking. Fish were captured once a week in non-consecutive weeks between the 14^th^ of October and the 18^th^ of November of 2016 using a low-voltage (400 V) electrofishing unit (Hans Grassl IG-200), according to European norms [71] and national guidelines [72]. No more than 80 fish were captured per sampling occasion. In total, 150 fish were captured (mean total length ± SD = 14.7 ± 1.6 cm). After electrofishing, fish were transported (50 minutes) in an aerated fish transport tank (Linn Thermoport 190 l) to the laboratory.

In the laboratory, the fish were distributed between two 900 l continuously aerated and biologically filtered holding tanks, and acclimated to ambient temperature and natural photoperiod for a 48–72 h period. Each holding tank was covered with a sunshade mesh, and clay roof tiles were placed in the bottom of the tanks to provide refuge areas. Water quality parameters (mean ± SD) were measured and adjusted in a daily basis using a multi parameter probe (YSI 556 MPS) for temperature (21.4 ± 1.7°C), pH (6.79 ± 0.28), dissolved oxygen (87.3 ± 5.8%) and conductivity (304.2 ± 38.6 μS.cm^-1^), and on a weekly basis using photometry (WTW- Spectroflex 6600) for nitrites (0.021 ± 0.004 mg.l^-1^) and ammonia (0.001 ± 0.001 mg.l^-1^). Partial water changes (15%) were performed every other day. The flume water temperature (24.0 ± 1.7°C) and dissolved oxygen (87.6 ± 6.4%) were monitored twice a day. The water quality parameters were in accordance with the national legislation for water quality standards to protect and improve the aquatic environment according to water use [73]. Fish were fed with a commercial diet for benthic species every night to avoid additional stress caused by food deprivation. Feeding took place only after the acclimation period.

### 2.3 Experimental facilities

**Fig 1:**
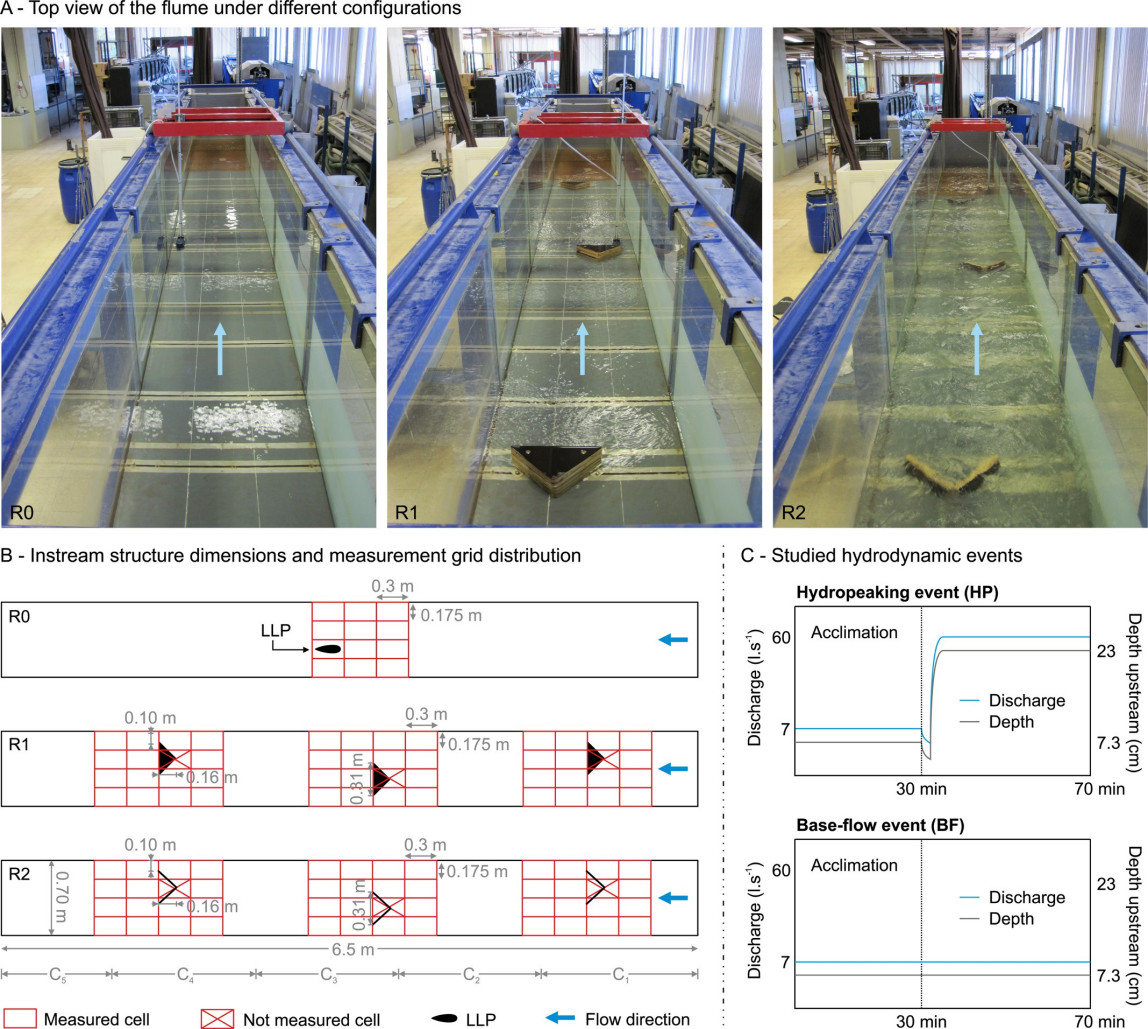
Summary of the experimental setup. A) Top view of the flume and test configurations (R0, R1 and R2). B) Plan of the setup with dimensions, grid of hydrodynamic measurements, and observation zones (C1 to C5). C) Depth and discharge of the studied hydrodynamic events.

The experiments were conducted between the 17^th^ of October and the 23^rd^ of November 2016 in an indoor flume located at the Laboratory of Hydraulics, at the University of Lisbon, Portugal (Fig 1). The flume has a rectangular cross-section, and is constructed on a steel frame with glass panels on both sides. The flume length was shortened to 6.5 m using two perforated metallic panels, and the flume width was 0.7 m (Fig 1). The discharge and the water level were controlled by a sluice gate upstream and by a flap gate downstream. The maximum discharge was 60 l.s^-1^. Two plywood instream structures were designed as potential refuges: solid triangular pyramids (R1) and v-shaped structures (R2), and compared with a configuration that did not have any instream structures (R0) (Fig 1).

Preferable substrate sizes should be at least in the same range as the fish length [74,75]. Thus, the refuge shape and dimensions of this study were chosen to have at least the same order of magnitude as the fish´s body length (~ 20 cm) [56]. The triangular shape creates a low velocity refuge immediately downstream of the structure and induces local flow areas in the wake with reduced vorticity [76]. Likewise, the total number of structures was based on the spatial density of refuges according to experimental results in fish passages [56]. The instream structures were placed in an alternating pattern, assuring that they were permanently wetted during base-flow conditions. This spatial arrangement intended to mimic a semi-natural displacement of substrate in the main river channel that may be exploited by fish for flow-refuging.

### 2.4 Hydrodynamic events

The flow ratio was 8.6, defined as the peak discharge (60 l.s^-1^) divided by the base-flow discharge (7 l.s^-1^) [77]. A flow ratio of this value is considered high for fish species according to the literature [41,77]. Two flow events were simulated; hydropeaking (HP) and base-flow events (BF) (Fig 1C). Before each event, *L*. *bocagei* acclimated in the flume for 30 minutes with a flow rate of 7 l.s^-1^ (Fig 1C). During this period, the flume upstream gate was left open at a 10° angle. The downstream gate was fixed at a 76° angle during all experiments. The HP event consisted of a single up-ramping event where *L*. *bocagei* were subjected to the peak discharge for 40 minutes after the acclimation period. To create this event, the upstream gate was shut completely while filling the flume reservoir. The discharge was manually controlled until attaining a flow rate of 60 l.s^-1^. Afterwards, the upstream gate was rapidly opened to 10° to release the peak flow and reach the constant flow regime. The mean time (± SD) for this procedure (up-ramping) was 28.12 ± 1.86 seconds, corresponding to an up-ramping rate of 0.55 cm.s^-1^ corresponding to a total increase of 15.7 cm in the water depth (rate of change) until reaching the constant flow regime. The BF event consisted of a continuous 7 l.s^-1^ discharge stimulus for 40 minutes corresponding to the absence of hydropeaking conditions. The combination of instream structures (i.e. R0, R1, and R2) with flow events (i.e. HP and BF) resulted in five different sets of experiments, as follows: R0HP, R1HP, R1BF, R2HP and R2BF.

For each event, a school of five fish was tested and replicated five times. Each fish was tested only once. It should be noted that larger groups of *L*. *bocagei* occur in nature, particularly during the reproductive season. A school size of five fish was selected to optimize the observation of fish movement behaviour in the flume. This was done in order to reproduce representative group behaviour and to reduce the number of captured fish.

### 2.5 Fish responses

#### 2.5.1 Physiological responses

To evaluate the potential physiological responses to hydropeaking between instream structure configurations (R0 *vs*. R1 and R2), the levels of blood glucose and lactate were quantified. These physiological parameters were chosen since glucose and lactate level increases are usually directly associated with primary responses to stress [24]. Additionally, changes in glucose and lactate levels have been widely used as secondary physiological indicators of stress to flow variability [31]. Lactate increases have been associated with prolonged anaerobic swimming when the organism can no longer maintain aerobic sustained swimming, resulting in exhaustion [78]. Hence, both physiological responses may represent reliable surrogates of a stress response to flow variability. After each flow event, each fish was dip-netted from the flume and transferred to a recipient with continuously oxygenated water and immediately placed in a v-shaped plastic trough in a supine position. Blood samples (0.1–0.5 ml) were collected via caudal puncture using 23 G or 25 G pre-heparinized needles. It has been demonstrated that three minutes is not long enough to have a significant influence on primary stress responses (e.g. cortisol) [79]. The glucose and lactate levels were immediately measured using the portable meters Accu-check Aviva (Roche) and Lactate Plus (Nova Biomedical UK) respectively. These lactate and glucose portable meters have been successfully tested and validated for fish research [80–82].

#### 2.5.2 Movement behaviour

**Fig 2:**
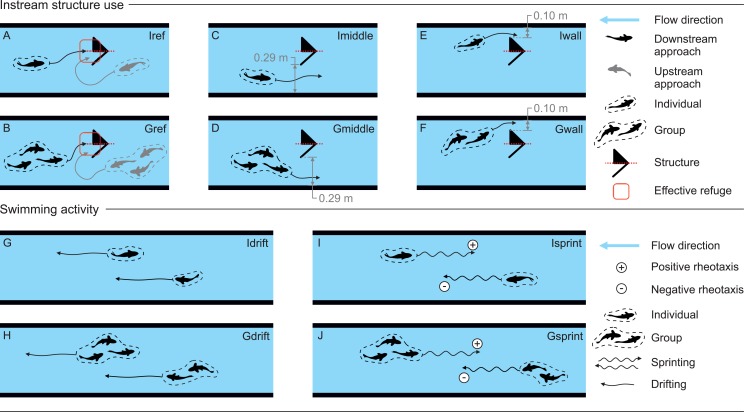
Classification of the behaviour metrics. (A and B)—Individual (Iref) and group (Gref) structure use; (C and D)–Individual (Imiddle) and group (Gmiddle) passage by the structures using longest distance between the structure and the flume wall; (E and F)–Individual (Iwall) and group (Gwall) passage by the structures using the shortest distance between the wall and the structure. (G and H) Individual (Idrift) and group (Gdrift) drifts. (I and J) Individual (Isprint) and group (Gsprint) sprints. Plus (+) and minus (-) signs represent the swimming direction of *L*. *bocagei*, as positive and negative rheotaxis respectively. These metrics were used for all configurations.

To examine the effects of instream structure type and flow event on *L*. *bocagei*, the behaviour metrics were selected based on fish body motion directed towards the structure and to changes in motion corresponding to the occurrence of a peak event. Afterwards, the behaviour metrics were divided into two categories: 1) structure use, and 2) swimming activity in the flume (Fig 2). The frequency of each behaviour was defined as the number of occurrences, in absolute frequency, over the duration of the flow event (i.e. 40 minutes). Each metric was attributed to a single fish (I) or to a group of two to five fish (G).

Successful structure use was considered when a single fish (Iref, Fig 2A) or a group of fish (Gref, Fig 2B) were observed in the immediate downstream area of the structure (Fig 2A and 2B). Considering the position of each structure in the flume, there were two possibilities for *L*. *bocagei* to pass by them and move upstream: by using the shortest (0.10 m) or the longest (0.29 m) distances between the structures and the correspondent flume wall (Fig 2C–2E). This positive rheotactic movement was registered when a single or a group of fish used either the shortest (Iwall, Fig 2E; Gwall, Fig 2F) or the longest (Imiddle; Fig 2C; Gmiddle, Fig 2D) distances respectively.

All swimming activity metrics were selected to represent movement responses which may be associated with the severity of a peak event. They were separated into two types; fish sprints, defined as a swimming activity lasting a few seconds and characterized by several tail beats (Isprint, Fig 2I, and Gsprint, Fig 2J), and fish drifts, defined as downstream fish displacements driven by passive advection of the body in the flow direction (Idrift, Fig 2G, and Gdrift, Fig 2H).

Activity metrics were counted considering the total flume usable area (observation zones C_1_ to C_5_, Fig 1B). A behaviour occurrence was only assigned to a specific observation zone only if it began at that location. Each individual or group were considered a sampling unit and any individual behaviour was registered if performed by one fish independently of the group.

### 2.6 Hydrodynamic characterization using an artificial lateral line

**Fig 3:**
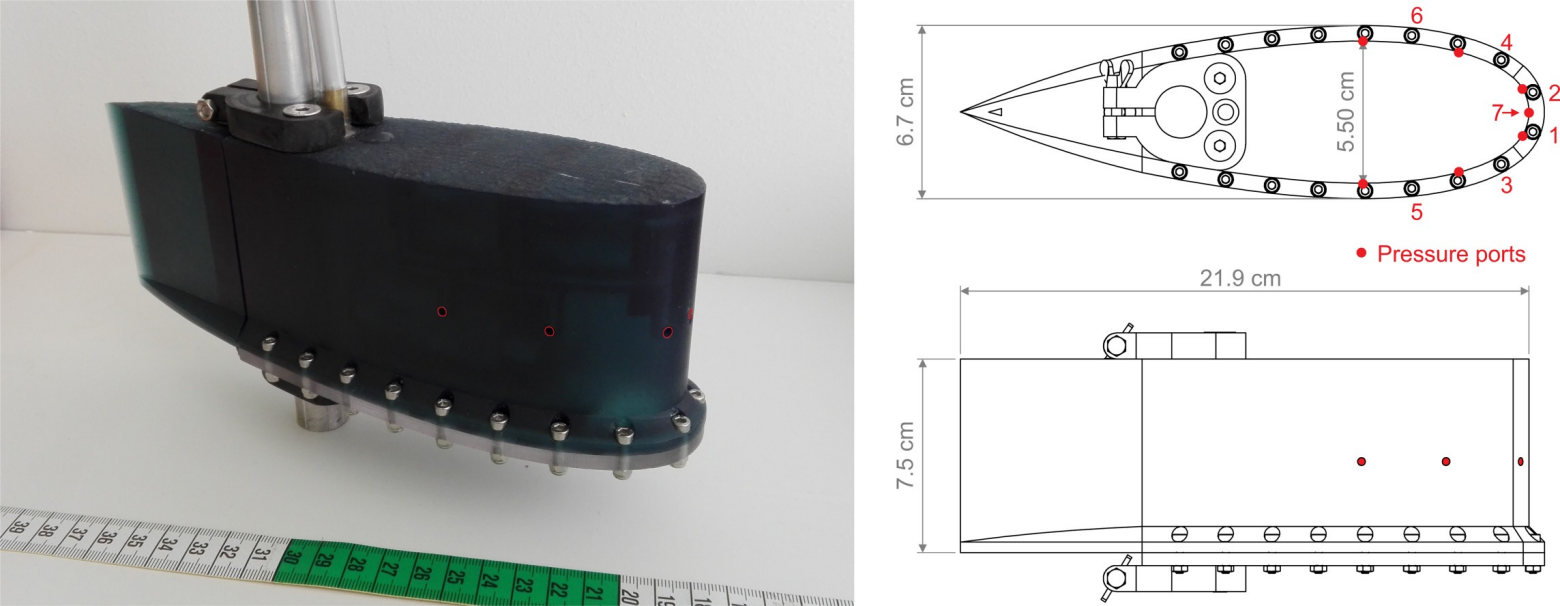
Lateral line probe (LPP). NACA025 body shape, showing the locations of the differential pressure sensors (1–6), and the absolute pressure sensor (7).

An artificial lateral line probe (LLP) was used to measure the local flow field properties during the different flow events and quantify the effects of instream structures. LLPs are streamlined bodies with a pressure sensitive sensor array. The devices allow for a characterization of the fluid-body interactions, and are capable of measuring at the same rates as the natural sensing frequencies of fish, from 10s to 100s Hz [83]. The LLP used in this study consisted of a 0.22 m length NACA025 body which measures pressure gradients over the body using six differential pressure sensors (±2000 Pa MPXV7002). All measurements with the LLP were conducted using a 200 Hz sampling rate. In addition, the water depth was measured by the probe using an absolute pressure sensor (0 to 10000 Pa—MPX5010GP) (Fig 3).

The selected variables were: mean front pressure, mean front fluctuations and mean pressure asymmetry (Table 1).

**Table 1:** Pressure-based variables form LLP measurements used in this study. Definition of mean pressure and mean pressure fluctuations are included to clarify the mathematical definition of the variables used for this study. n: number of data points in a measurement; i: index of the sensor.

Variables	Equation
Mean pressure	${\bar{p}}_{i}=\frac{\sum_{j=1}^{n}p_{i,j}}{n}$
Mean pressure fluctuations	$\bar{p}{'}_{i}=\frac{\sum_{j=1}^{n}\left|p_{i,j}-{\bar{p}}_{i}\right|}{n}$
Mean front pressure (${\bar{p}}_{12}$)	${\bar{p}}_{12}=\frac{\sum_{j=1}^{n}\frac{p_{1,j}+p_{2,j}}{2}}{n}$
Mean front pressure fluctuations ($\bar{p}{'}_{12}$)	$\bar{p}{'}_{12}=\frac{\bar{p}{'}_{1}+\bar{p}{'}_{2}}{2}$
Mean pressure asymmetry ($\Delta {\bar{p}}_{1-6}$)	$\Delta {\bar{p}}_{1-6}=\frac{\sum_{k=1}^{m}\frac{\sum_{j=1}^{n}{\left(p_{2k-1,j}-p_{2k,j}\right)}^{2}}{n}}{3}$

The mean front pressure (${\bar{p}}_{12}$) is quadratically related to the velocity of the flow, following the conservation of energy [84]. This variable is also directly correlated with the free stream flow velocity experienced by the fish body. Higher mean front pressure magnitudes also imply an increase in the hydrodynamic drag, thus upstream movements may be reduced and increase the fish energy consumption during station holding [85]. The mean front pressure fluctuations ($\bar{p}{'}_{12}$) represent the magnitude of the rapid changes of pressure gradients on the body of the LLP, and therefore, they are correlated with the turbulence experienced by the probe [86]. They represent the changes in pressure magnitudes over time at a given location. In general, lower pressure magnitudes correspond to regions of uniform and stable flow conditions. Fish, and in particular *L*. *bocagei*, have shown preferences for low turbulence areas [60,87]. The mean pressure asymmetry ($\Delta {\bar{p}}_{1-6}$) offers an instant comparison of the pressure gradient differences in each side of the LLP. This allows the identification of cyclic flow patterns (e.g. repeating patterns of vortices) and their magnitude [88]. It is also worth noting that vortices of a scale in the same order of magnitude as a fish’s body length may also be utilized to reduce swimming cost and can even be leveraged to enhance upstream movements [89].

The selected measurement grid (Fig 1), covered the regions most affected by the rapid flow changes as well as the peak discharge for R1 and R2. The grid was broken into volume elements whose lateral and streamwise dimensions closely match the body length of young adults of *L*. *bocagei* as well as the LLP.

### 2.7 Data analysis

A Kruskal-Wallis analysis was performed to verify whether the hydrodynamic event and structure type/presence triggered physiological responses in *L*. *bocagei*. The analysis determined if there were statistical differences in the levels of blood glucose and lactate among replicates of each event. As there was not any statistical evidence that supported that hypothesis, the lactate and glucose levels of each *L*. *bocagei* were considered as true replicates. Afterwards, to verify if there were any significant differences in the levels of blood glucose and lactate of *L*. *bocagei* among events, a Kruskal-Wallis test was applied with a Nemenyi post-hoc test for pairwise contrasts [90]. Post-hoc tests for pairwise contrasts between events were made using the R-package PMCMR [90].

To provide a visual representation of the main trends for instream structure use (i.e. Iref, Gref, Imiddle, Gmiddle, Iwall, Gwall) and swimming activity (Isprint, Gsprint, Idrift, Gdrift) of *L*. *bocagei* according to the hydrodynamic events, a correspondence analysis (CA) was conducted. This method preserves the χ2 distance among objects, does not require data normalization, and it is suitable for frequency-like data [91]. CA is adequate for this study due to the nature of the behaviour response metrics (absolute frequency) and its reduced number (i.e., six for instream structure use, and four for swimming activity) [91]. This analysis was conducted using the R-package vegan [92]. To test if the event was affecting 1) the frequency of instream structure use and 2) the swimming activity of *L*. *bocagei* the two-way distance-based multivariate analysis of variance was applied (using Euclidean distance and 999 permutations) [93]. This method does not require the assumptions of parametric tests [94], is suitable for small sample sizes [95] as well as continuous and factor predictors [93]. The analysis was conducted using the R-package vegan [92]. If an effect was detected, a detailed analysis per metric of refuge use and flume swimming activity was conducted by Kruskal-Wallis analysis with a post-hoc Nemenyi test for pairwise contrasts between events [90]. This analysis was conducted using the R-package PMCMR [90]. All statistical analyses were performed using R version 3.3.2 [96].

The variables measured with the LLP were plotted onto the grid to illustrate and compare the differences among the studied events. A summary of the measured minimum values of the pressure variables in the area affected by the structures and the mean ± *SD* values in the flume area were considered. This was performed to simplify the discussion of the LLP results, compare relationships to the fish movement responses, and define local flow preferences for this fish species. The area affected by the structure consisted of 3 grid rectangles measured downstream and near the structure (3 rectangles behind each structure, n = 9), and the flume area consisted of the remaining grid rectangles (n = 45–9 = 36). All data analysis, plots, and calculations concerning LLP were performed using MATLAB release R2017a.

## 3. Results

### 3.1 Physiological responses

**Fig 4:**
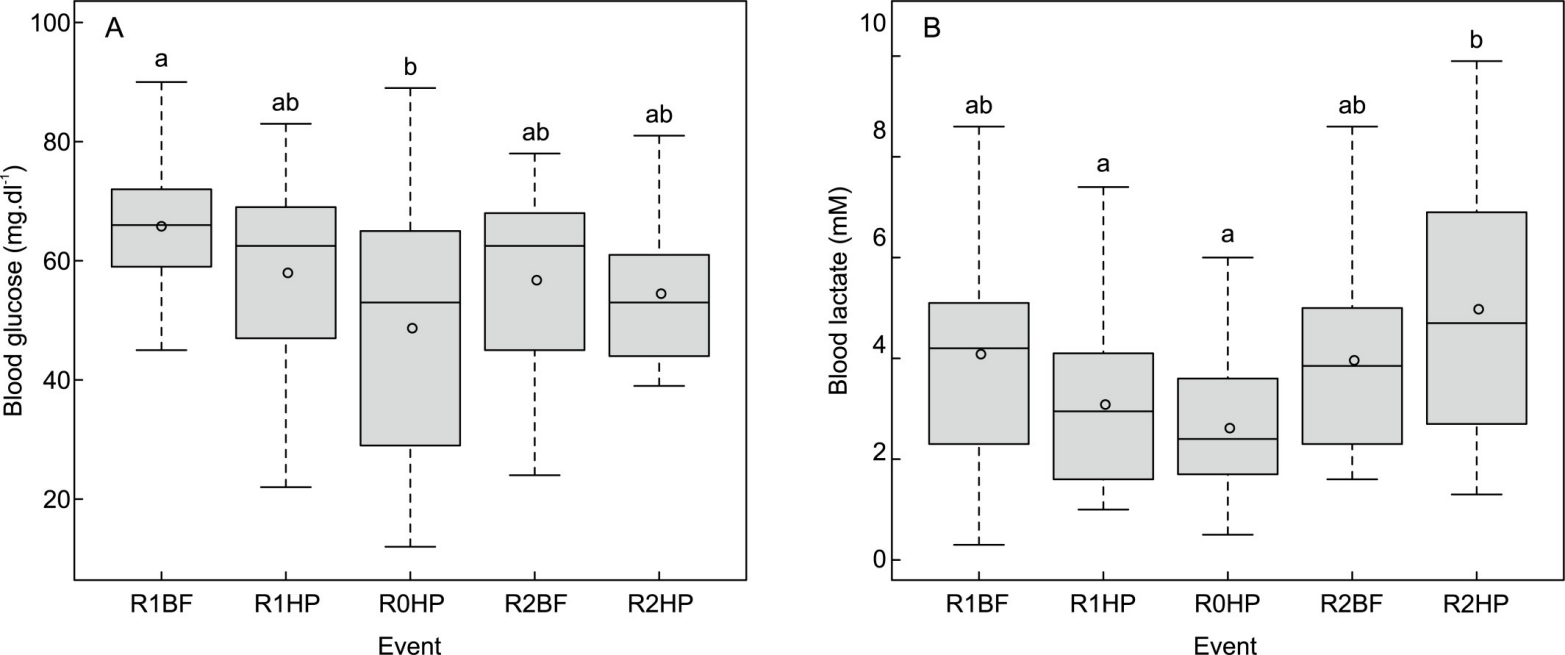
**Boxplots of the variation of (A) blood glucose (mg.dl**^**-1**^**) and (B) lactate (mM) levels for *L*. *bocagei*.** R1BF and R2BF–Base-flow event in the presence of solid triangular pyramids and v-shaped structures respectively; R0HP, R1HP and R2HP–Hydropeaking event in the absence of structures, presence of solid triangular pyramids and the presence of v-shaped structures, respectively. The letters correspond to the post-hoc test results. The circles correspond to the mean value of the physiological response for each treatment.

Glucose levels differed significantly among events (χ2 (4) = 10.870, *p* = 0.028) and were higher in *L*. *bocagei* subjected to R1BF when compared to R0HP (χ2 = 4.331, *p* = 0.019) (Fig 4A). The lactate levels differed significantly among events (χ^2^ (4) = 17.141, *p* = 0.002) and were higher in R2HP compared to R0HP (χ^2^ = 5.117, *p* = 0.003) and R1HP (χ^2^ = 4.161, *p* = 0.027) (Fig 4B).

### 3.2 Movement behaviour

**Fig 5:**
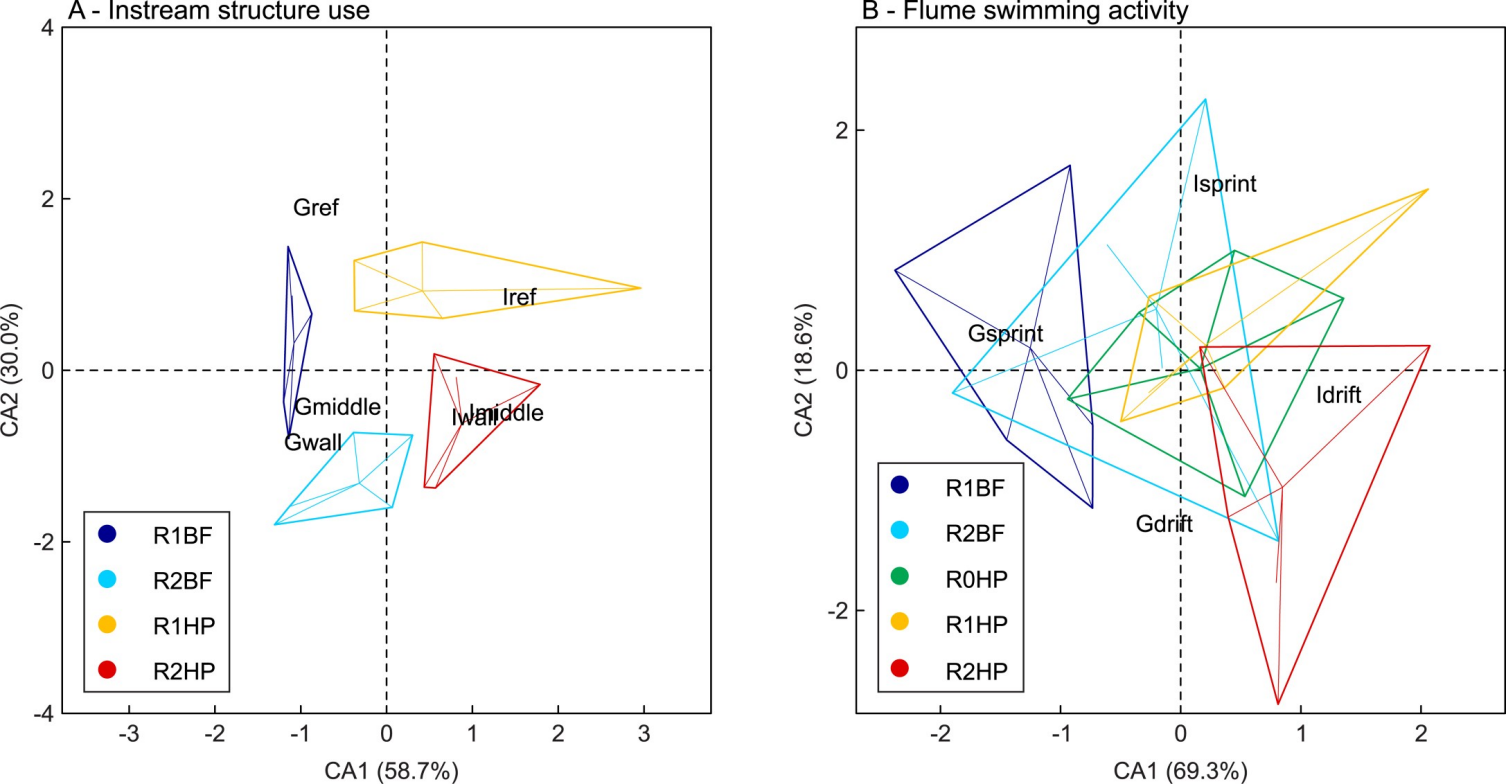
**Correspondence analysis (CA) biplots for the instream structure use (A) and swimming activity in the flume (B).** The biplots illustrate the spatial distribution of behaviour according to each flow event. The axes labels indicate the proportion (%) attributed to the spatial distribution of the events. R1BF and R2BF–Base-flow event in the presence of solid triangular pyramids and v-shaped structures respectively; R0HP, R1HP and R2HP–Hydropeaking event in the absence of structures, presence of solid triangular pyramids and presence of v-shaped structures respectively.

The CA orthogonal axes represent the main trends observed for the frequency of instream structure use and swimming activity in the flume according to the hydrodynamic events (Fig 5). The first two axes for the frequency of structure use explain 88.7% of the variation among events (Fig 5A). BF opposes HP on the first CA axis, whereas R1 opposes R2 on the second CA axis (Fig 5A). A clear trend can be observed considering group structure use in BF in opposition to individual structure use in HP (Fig 5A). The spatial organization and the structure use scores, indicated that *L*. *bocagei* individuals more frequently make use of R1, particularly in R1HP. The path chosen to cross the structures was more evident in the R2HP, particularly for individuals as well (Fig 5A). The first two axes for the swimming activity account for 87.9% of the variation among events (Fig 5B). R1BF opposes to R2HP in the first CA axis for group *vs*. individual behaviour respectively (Fig 5B). Gsprint was the most frequent behaviour in R1BF, whereas Idrift was the most frequent behaviour in R2HP (Fig 5B). In the absence of instream structures, i.e. in R0HP, there was a more evenly distributed swimming activity pattern.

**Fig 6:**
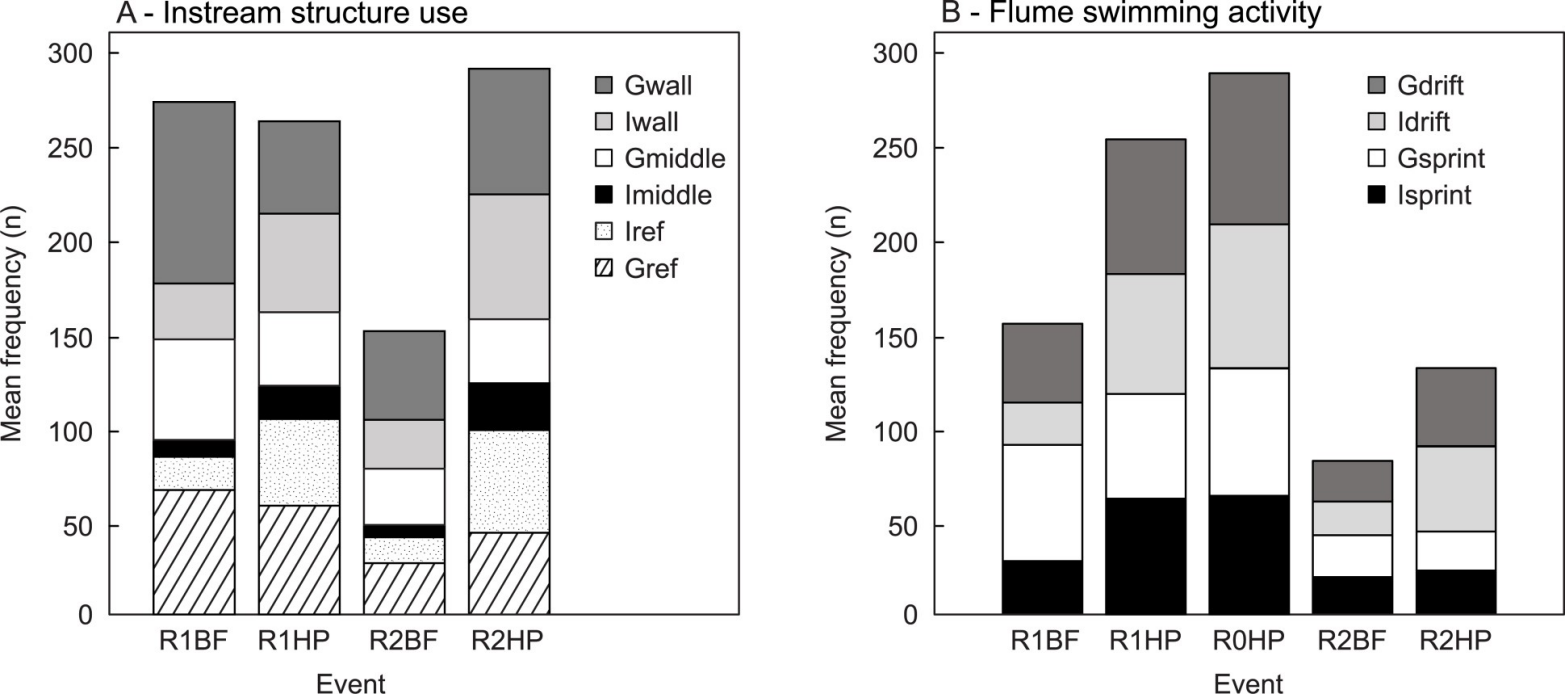
Mean frequency (n) of each behaviour metric for each event. A) Structure use (Iref and Gref), path chosen to cross the structures (Imiddle, Gmiddle, Iwall, Gwall). B) Flume swimming activity (Isprint, Gsprint, Idrift and Gdrift). R1BF and R2BF–Base-flow event in the presence of solid triangular pyramids and v-shaped structures respectively; R0HP, R1HP and R2HP–Hydropeaking event in the absence of structures, presence of solid triangular pyramids and presence of v-shaped structures respectively.

The ordination results were supported by the multivariate analysis, where a significant effect of flow event on the structure use (F = 5.156, *p* = 0.001) and swimming activity (F = 7.839, *p* = 0.001) of *L*. *bocagei* was found. There were significant differences in the mean frequency of individual (Iref; χ^2^ (3) = 11.271, *p* = 0.010) and group structure use (Gref; χ^2^ (3) = 12.233, *p* = 0.007) among flow events. Rank comparisons showed that Iref was higher for *L*. *bocagei* that were subjected to R1HP and R2HP in comparison with R2BF (*p* = 0.038 and p = 0.033 respectively) (Fig 6A). Groups of *L*. *bocagei* used the downstream area of solid triangular pyramids more frequently in the base-flow (R1BF) and in the hydropeaking events (R1HP) in comparison with the base-flow in the presence of v-shaped structures (R2BF) (*p* = 0.008 and *p* = 0.044 respectively) (Fig 6A).

There were also significant differences in the path that fish selected to cross the structures for individual behaviour (Imiddle; χ^2^ (3) = 9.398, *p* = 0.024; Iwall; χ^2^ (3) = 8.504, *p* = 0.037), but not for group behaviour (Gmiddle: (χ^2^ (3) = 5.597, *p* = 0.133; Gwall: (χ^2^ (3) = 7.563, *p* = 0.056). The mean frequency of Imiddle was significantly higher for R2HP in comparison with R2BF (F = 3.817; *p* = 0.035) (Fig 6A).

The mean frequency of flume swimming activity differed significantly among flow events and structure configurations for Isprint (χ^2^ (4) = 16.525, *p* = 0.002), Gsprint (χ^2^ (4) = 13.374, *p* = 0.010), Idrift (χ^2^ (4) = 18.49, *p* = 0.001) and Grift (χ^2^ (4) = 15.403, *p* = 0.004). The mean frequency of Isprint was significantly higher in R0HP (F = 4.375; *p* = 0.017) and R1HP (F = 4.010; *p* = 0.037) in comparison with R2BF (Fig 6B).The mean frequency of Idrift was significantly higher in R0HP (F = 4.466, *p* = 0.013) and R1HP (F = 3.859, p = 0.049) in comparison with R1BF, and in R0HP (F = 4.618, p = 0.010) and R1HP (F = 4.010, p = 0.037) in comparison with R2BF (Fig 6B). The mean frequency of Gdrift was significantly higher in R0HP (F = 4.739, *p* = 0.007) and R1HP (F = 4.618, *p* = 0.010) in comparison with R2BF (Fig 6B).

Three major trends were apparent after integrating the physiological and movement behaviour metrics: 1) Lower levels of glucose in R1BF were associated with an increased group structure use; 2) Higher lactate levels in R2HP where associated with increased individual attempts to cross the structures and move upstream; 3) The absence of significant differences in the levels of both glucose and lactate in R1HP and R0HP was associated with an increased structure use in R1HP, and in the drift frequency in R1HP and R0HP (Table 2).

**Table 2:** Summary of the comparisons of the physiological and behavioural responses of *L*. *bocagei* between events tested. The direction of physiological responses is indicated (↑ higher, ↓ lower). The frequency of the shown movement behaviour metrics was higher in that event in comparison to those indicated (number).

Event	*L*. *bocagei* responses		
	Physiology	Movement behaviour	
		Structure use	Swimming activity
R1BF	↓Glucose^1^	Gref^3^	
R1HP		Gref^3^; Iref^3^	Isprint^3^; Idrift^4^; Gdrift^3^
R0HP		N/A	Isprint^3^; Idrift^4^; Gdrift^3^
R2BF			
R2HP	↑Lactate^2^	Iref^3^; Imiddle^3^	

^1^ In comparison with R0HP;

^2^ in comparison with R1HP and R0HP;

^3^ in comparison with R2BF;

^4^ in comparison with R1BF and R2BF; N/A not applicable

### 3.3 Hydrodynamic characterization using an artificial lateral line

**Fig 7:**
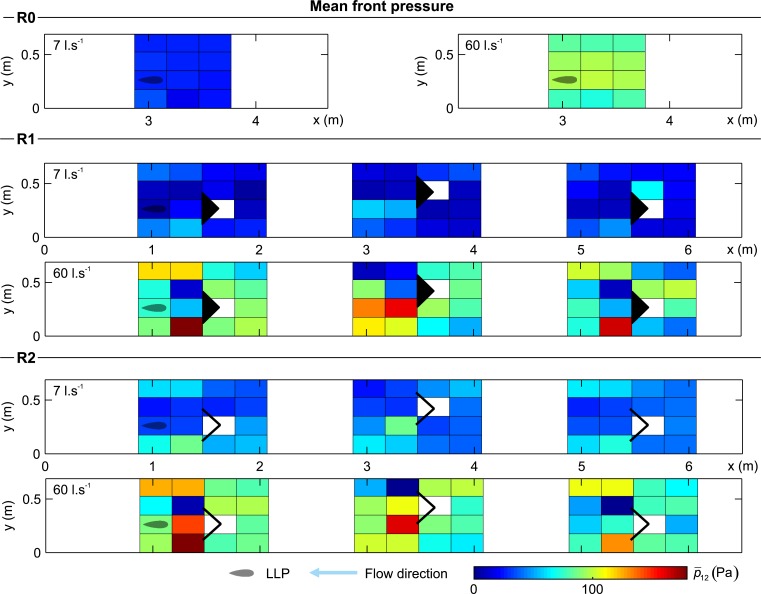
Distribution of mean front pressure (${\bar{p}}_{12}$) for all configurations and both discharge scenarios considered (7 and 60 l.s^-1^). The reader is referred to supporting information (S1 Fig) to look at the contour representation of this figure.

**Fig 8:**
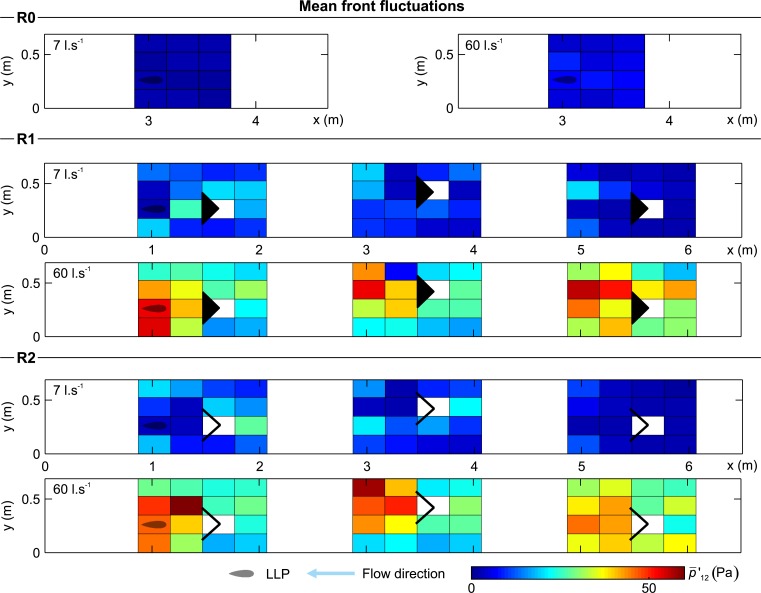
Distribution of mean front fluctuations ($\bar{p}{'}_{12}$) for all configurations and both discharges scenarios considered (7 and 60 l.s^-1^). The reader is referred to supporting information (S2 Fig) to look at the contour representation of this figure.

**Fig 9:**
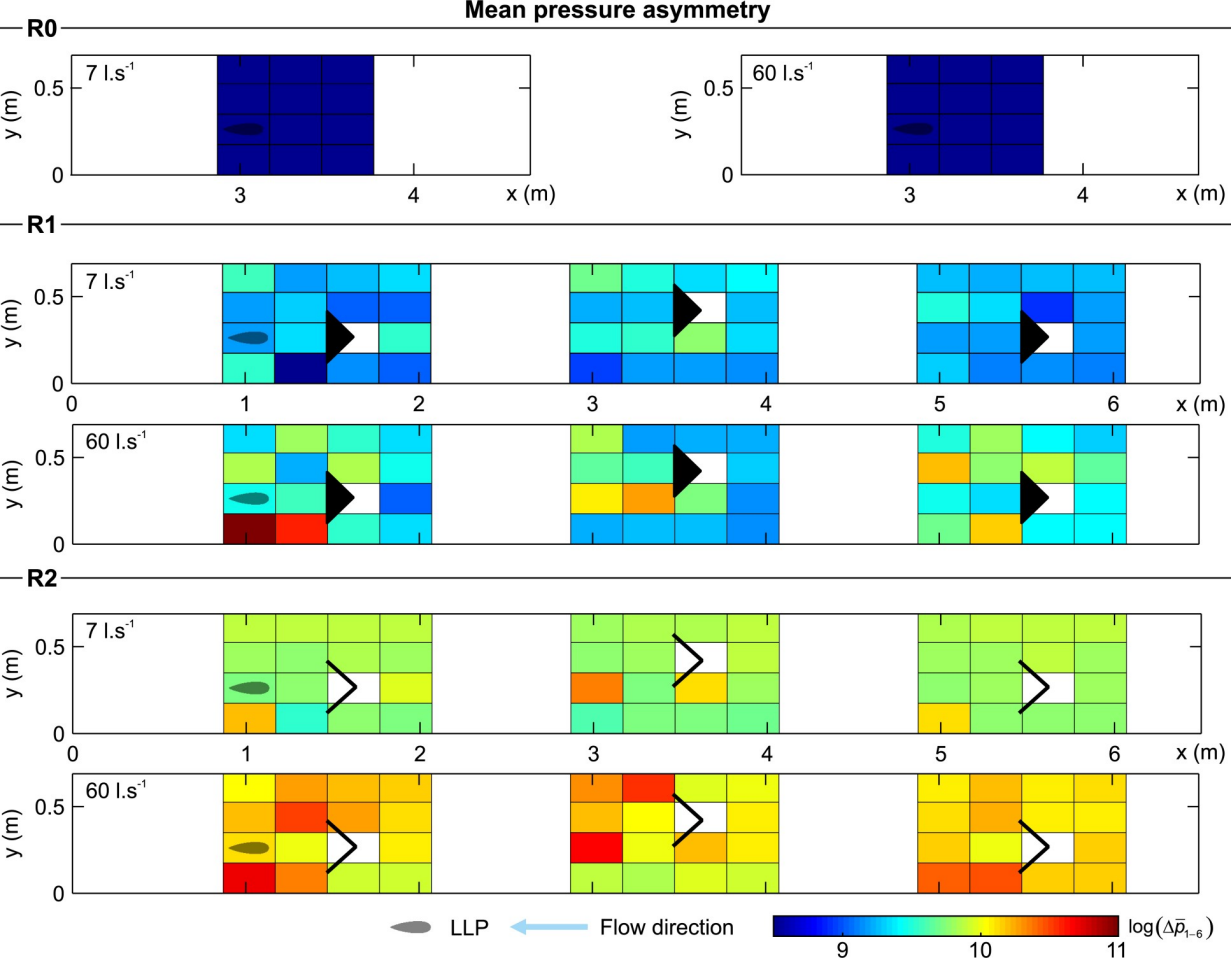
Distribution of mean pressure asymmetry ($\Delta {\bar{p}}_{1-6}$) for all configurations and both discharges scenarios considered (7 and 60 l.s^-1^). The reader is referred to supporting information (S3 Fig) to look at the contour representation of this figure.

The distribution of mean front pressure represents the total mean pressure difference experienced by the LLP in the front of its body (${\bar{p}}_{12}$, Fig 7). Figs 7, 8 and 9 provide a representation of the measured pressure-based variables considered in this study:${\bar{p}}_{12}$, $\bar{p}{'}_{12}$ and $\Delta {\bar{p}}_{1-6}$. It was found that for all configurations (R0, R1 and R2) that an increase in discharge leads to higher values of ${\bar{p}}_{12}$. In the absence of structures, the discharge distributed nearly uniformly over the cross-section and ${\bar{p}}_{12}$ increased solely as a function of discharge (R0, Fig 7). However, the presence of structures (R1 and R2) will generate a low ${\bar{p}}_{12}$ area immediately behind the structure (refuge effect), and areas of higher ${\bar{p}}_{12}$ occur due to a local flow acceleration adjacent to the structure (Fig 7 and Table 3). The distribution of these flow-refuging areas downstream the structure is driven by the flow direction.

**Table 3:** Minimum (Pa) and mean ± *SD* (Pa) LLP derived pressure variables. The minimum pressure values (Pa) for mean front pressure (${\bar{p}}_{12}$), mean front pressure fluctuations ($\bar{p}{'}_{12}$) and mean pressure asymmetry ($\Delta {\bar{p}}_{1-6}$) refer to the results observed in the three rectangles measured behind the structure, Mean ± SD (Pa) refer to the pressure values observed in the flume (excluding structure area) for events R1BF, R1HP, R0HP, R2BF and R2HP.

Tested event	LLP derived pressure variables					
	Structure area (minimum)			Flume (mean ± *SD*)		
	Front pressure (Velocity) (Pa)	Front fluctuations (Turbulence) (Pa)	Asymmetry (log Pa)	Front pressure (Velocity) (Pa)	Front fluctuations (Turbulence) (Pa)	Asymmetry (log Pa)
R1BF	5.70	1.72	8.39	23.33±14.17	9.04±6.19	9.27±0.19
R1HP	11.18	6.52	9.17	80.32±24.78	30.62±11.55	9.55±0.38
R0HP	-	-	-	87.21±9.92	18.13±1.92	2.08±0.01
R2BF	30.35	1.51	9.54	44.15±12.13	9.66±7.02	9.86±0.14
R2HP	-4.88	31.16	10.00	84.39±18.29	30.80±10.18	10.14±0.19

The mean front pressure fluctuations quantify the change of the pressure magnitude over time, with lower values indicating more stable zones ($\bar{p}{'}_{12}$, Fig 8). In the absence of structures $\bar{p}{'}_{12}$ presented a uniform distribution driven only by the discharge and flume geometry (Fig 8). The presence of the structure generated a region of high-pressure fluctuations, $\bar{p}{'}_{12}$ (area of high turbulence) (R1 and R2, Fig 8 and Table 3). The distance to this highly fluctuating area depended on the discharge. Lower fluctuations were found near, inside and immediately behind the structure (refuge effect), and between structure and walls due to the coherence created by converging streamlines.

The mean pressure asymmetry represents the instantaneous pressure differences between both sides of the probe, detecting and quantifying the vorticity experienced ($\Delta {\bar{p}}_{1-6}$, Fig 9). Similar to the other variables, its distribution shows that in the absence of structures, the asymmetry remains uniform and with low magnitude. This is primarily due to the absence of planar vortices shed by the structures (R0, Fig 9). In general, it was observed that $\Delta {\bar{p}}_{1-6}$ had a larger in R2 than R1, and increased slightly as a function of discharge.

## 4. Discussion

The effects of simulated hydropeaking events including instream structures as flow refuges for *L*. *bocagei* were investigated. A novel, interdisciplinary approach combined physiological and behavioural responses with fluid-body interactions measured with a fish-inspired artificial lateral line. The combinations of hydrodynamic events and instream structures altered the spatio-temporal distribution and magnitude of velocity, turbulence fields and pressure fluctuations (Figs 7–9), and were found to correspond to distinct physiological and behavioural responses (Figs 4–6 and Table 2).

The physiological and behavioural responses differed between hydropeaking and base-flow conditions as well as with instream structure configuration. An interesting finding was that although flow and behaviour were indeed linked, the extreme hydrodynamic changes did not always result in higher physiological levels or frequencies in movement behaviour (Fig 6 and Table 2). Similarly, in rivers affected by hydropeaking, there were no significant physiological increments in salmonids possibly explained by flow-refuging, feeding and social interactions [19]. Although higher swimming costs are associated to high flows, fish can use these as migratory and spawning cues [42], and use the low flows to optimize foraging behaviour [97]. As an example, glucose and lactate levels were not systematically higher considering HP events. The results indicate that *L*. *bocagei* responses may be related to the combination of flow event severity and structure configuration, rather than solely to the severity of the flow event itself. Given this evidence, the lowered physiological responses and frequencies of movement behaviour observed in R2BF (particularly in comparison with R1BF) and the increased structure use and drift frequency (as a means to recover from the peak-flow conditions) in R1HP indicate that these event-configuration combinations can be favourable for *L*. *bocagei*.

The highest lactate levels were observed in R2HP. This can be explained by the higher frequency of passages observed in R2 (Fig 6A) together with the lower frequency of drifts (Fig 6B). An increase in observed swimming velocities corresponds to higher swimming costs [98,99], indicating that the fish had difficulty maintaining their focal position while crossing the structures, which may explain the higher lactate levels. In addition, the lower frequency of drifts observed in R2HP when comparing with R0HP and R1HP (Fig 6B), and the higher lactate levels (Fig 4B) may indicate that the thresholds of pressure variables during this event, and in particular asymmetry (Fig 9), may have acted to hinder fish drifts. Although drifts are typically associated with the inability to hold position, the lactate elevations denote increasing swimming effort. These results suggest that the hydrodynamics shed by the v-shaped structures could have hindered the use of these structures as flow-refuging areas. Furthermore, the observed drifting behaviour may provide a means to recover from the additional swimming effort. This possibility is also supported by the lower levels of lactate and the identical frequencies of drifts and sprints in R0HP and R1HP (Fig 6B). Still, when subjected to R2HP, *L*. *bocagei* were attracted to the side of the structures near the flume wall (Fig 6A), which may correspond to areas of reduced locomotion effort. It is well-established that fish can take advantage of the velocity, turbulence, size and periodicity of vortices (Figs 7–9) [100–102]. This is because as undulatory swimmers, fish are able to take advantage of the interactions between their body and the local hydrodynamic environment [101]. In nature, these individual interactions result from the existing physical properties such as flow fluctuations present in geometrically heterogeneous habitats. For habitat mitigation purposes, heterogeneity should be considered as a crucial design parameter, while carefully considering the local hydrodynamic conditions, otherwise it may produce the opposite effect [103]. Indeed, it is established that fish require a wide range of structures for refuge. Fish have been observed avoiding rapid flow conditions by choosing low flow areas for flow-refuging [16,18,19,26,47], hiding in available rocky substrates [44], and using lateral refuge [43].

Despite the higher lactate levels observed in R2HP, there was no visible indication that *L*. *bocagei* were exhausted, as the fish were able to maintain the burst swimming during the total duration of the event. Mean lactate levels found in a fish passage study, where *L*. *bocagei* was subjected to a 110 l.s^-1^ for 90 minutes, were higher (6.69±1.95 mM) than those registered in this study (5.06±0.49 mM)[62]. Thus, the results from this study may reflect physiological adjustments for *L*. *bocagei* to cope with the created hydrodynamic changes. Considering glucose, previous studies have shown increased levels for *L*. *bocagei* subjected to the most extreme hydrodynamic changes [27]. In this study, no clear association was found.

The lowest movement behaviour frequency observed in R2BF suggests that this event-configuration is the most beneficial for *L*. *bocagei*. The lower frequency of drifts observed for individuals and groups suggests that this combination favoured both *L*. *bocagei* to hold station without significant energy expenditure (Fig 6B and Table 3). On the other hand, the reduced frequency of structure use may indicate that the hydrodynamic conditions created in the flume area were not adverse enough to induce refuge use (Fig 6A and Table 3). For all studied configurations under HP, the observed drifts were most commonly associated to a higher frequency of sprints (Fig 6B and Table 3). This could be motivated by an exploratory behaviour to find suitable flow areas. For example, when brown trout were exposed to fluctuating flows (repeated up- and down-ramping events) the possibility to hide in available substrate denoted a compensatory behaviour, where after being subjected to repeated stimuli brown trout was able to adjust both physiologically (return of cortisol to pre-stress levels) and behaviourally (hiding in substrate) to avoid negative effects [16]. This compensatory behaviour suggests an adaptive mechanism for juvenile brown trout to re-establish homeostasis [16,104]. In this study, it was found that the absence of such suitable areas motivated upstream sprinting, by crossing the structures or sprinting upstream to negotiate with the created flow conditions. Sprinting behaviour was more evident for *L*. *bocagei* during R2HP. These results may indicate that the severity of the flow event together with the presence of R2 may have not provided sufficient low flow areas to recover, forcing individual behaviour. Conversely, the hydrodynamic conditions in R0 and R1 seemed to induce drifting (Fig 6B and Table 3). This could reflect an adaptive behaviour to recover from the effort of sprinting as a means to conserve energy and may explain the lower lactate levels observed for R1HP and R0HP (Fig 4A and 4B).

The differences observed in individual and group behaviour between HP and BF (Fig 5A and 5B) show that the changing flow conditions were not always favourable to maintain schooling. Although fish in groups usually benefit from the increased tail beat frequency of the leading fish [101] the existing flow conditions under HP were not always favourable. Group dynamics were particularly marked under R1BF indicating that the lower discharge and pressure magnitudes favoured group stability. The advantages of schooling behaviour have been demonstrated to reduce the total swimming costs under turbulent flows [105] and during reproductive migrations [106,107].

From the analyses of the physiological and behavioural results together with the artificial lateral line probe measurements, the asymmetry was the most related with behavioural observations. This was particularly evident in the two extremes R2BF and R2HP. Asymmetry, when used as a local flow variable enabled a comparison of the cycles of large-scale vorticity among events, which fish may use to reduce the costs for swimming [101]. Considering the different flow event and structure trials, a “favourable asymmetry window” was observed in R2BF for *L*. *bocagei* (Fig 9 and Table 2). This critical threshold for asymmetry corroborates the third hypothesis, that local hydrodynamic variables exist generating unique movement behaviour patterns. This was particularly evident for R2BF and R2HP (Fig 9). The thresholds for the pressure variables observed in R1HP and R0HP (Table 3) resulted in similar frequencies of sprints and drifts (Fig 6B). These results may denote drifting as a compensatory behaviour to the effort required to sprint. It has been suggested that in rivers affected by hydropeaking fish may change their position by moving backwards and forward between areas with suitable velocity and depth to avoid the negative effects of flow fluctuations [16].

Each event, HP and BF, combined with R1 or R2, generated unique local flow conditions which were more or less favourable for fish. Considering this, a geometrically heterogeneous configuration of instream structures (i.e. different shapes and sizes) could increase the probability for fish to find suitable refuge areas. As demonstrated, in the absence of suitable flow areas or under hydropeaking conditions, fish benefit from the presence of instream structures for flow-refuging. Nonetheless, complex habitats may also create unfavourable hydraulic conditions for fish [28,55]. Experimental concepts and engineering design of instream mitigation structures, should therefore take into account the interaction between channel morphology and water level changes [12].

## 5. Conclusions

This study provides experimental evidence that rapidly changing flow events in the presence of instream structures can trigger differentiated physiological and behavioural responses in *L*. *bocagei*. The LLP results further demonstrated that the combination of flow events with instream structures resulted in unique flow conditions, characterized by distinct velocity, turbulence and vorticity distributions in the flume, and particularly near the structure area. As these hydrodynamic features were unique to each combination, and specific behavioural responses were found, critical thresholds of local hydrodynamic variables for *L*. *bocagei* were defined accordingly (Table 3). In summary, under HP conditions lower velocities and higher turbulence and vorticity promote individual structure use, whereas higher velocities and milder turbulence and vorticity were found to promote sprints and drifts. Under BF conditions, the more frequent group behaviour and the lower refuge use, indicate that milder velocities in the structure area and lower mean turbulence and vorticity in the remaining flume area create favourable flow conditions for *L*. *bocagei*.

Finally, lower discharge magnitudes, and milder flow-ratios (< 8.6), are advantageous for this species and have the potential to reduce the energetic costs associated with rapid flow fluctuations. However, the simultaneous absence of a glucose response and exhaustion signs when *L*. *bocagei* were subjected to 60 l.s^-1^ encourage further investigation to understand the extent to which flow event-configurations combinations in rapid flow fluctuations trigger physiological responses in *L*. *bocagei*. Additionally, combined effects which consider the range of environmental factors that fish experience *in situ*, such as thermopeaking, are worthy of further investigation. To benefit from the individual interactions with the velocity, turbulence and vorticity of the hydrodynamic environment, diverse sets of instream structures should be preferred as potential flow-refuging areas for *L*. *bocagei* subjected to hydropeaking. Further research is encouraged to establish a mechanistic and quantifiable link between the derived pressure variables and specific fish responses, particularly the ability of fish to demonstrate adaptive mechanisms (e.g. compensation by searching favourable pressure thresholds) to avoid the negative effects of rapid flow fluctuations.

## Supporting information

S1 FigContour representation of mean front pressure (${\bar{p}}_{12}$).(EPS)

S2 FigContour representation of mean front fluctuations ($\bar{p}{'}_{12}$).(EPS)

S3 FigContour representation of mean pressure asymmetry ($\Delta {\bar{p}}_{1-6}$).(EPS)

S1 File *L*. *bocagei* physiological and behavioural data collected. (XLSX)

S2 File Data obtained from the fish-inspired lateral line probe (LLP). (XLSX)
